# Evolutionary Phylodynamics of Korean Noroviruses Reveals a Novel GII.2/GII.10 Recombination Event

**DOI:** 10.1371/journal.pone.0113966

**Published:** 2014-12-12

**Authors:** Thoi Cong Truong, Van Thai Than, Wonyong Kim

**Affiliations:** Department of Microbiology, Chung-Ang University College of Medicine, Seoul, South Korea; Tulane University, United States of America

## Abstract

Viral gastroenteritis is the most common causal agent of public health problems worldwide. Noroviruses cause nonbacterial acute gastroenteritis in humans of all ages. In this study, we investigated the occurrence of norovirus infection in children with acute gastroenteritis admitted to university hospitals in South Korea. We also analyzed the genetic diversity of the viruses and identified novel recombination events among the identified viral strains. Of 502 children with acute gastroenteritis admitted to our three hospitals between January 2011 and March 2012, genotyping of human noroviruses was performed in 171 (34%) norovirus-positive samples. Of these samples, 170 (99.5%) were in genogroup II (GII), while only one (0.5%) was in genogroup I (GI). The most common GII strain was the GII.4-2006b variant (n = 96, 56.5%), followed by GII.6 (n = 23, 13.5%), GII.12 (n = 22, 12.9%), GII.3 (n = 20, 11.8%), GII.2 (n = 6, 3.5%), GII.b (n = 2, 1.2%), and GII.10 (n = 1, 0.6%). Potential recombination events (polymerase/capsid) were detected in 39 GII strains (22.9%), and the most frequent genotypes were GII.4/GII.12 (n = 12, 30.8%), GII.4/GII.6 (n = 12, 30.8%), GII.4/GII.3 (n = 8, 20.5%), GII.b/GII.3 (n = 3, 7.7%), GII.16/GII.2 (n = 2, 5.1%), GII.4/GII.2 (n = 1, 2.6%), and GII.2/GII.10 (n = 1, 2.6%). For the first time, a novel GII.2/GII.10 recombination was detected; we also identified the GII.16/GII.2 strain for the first time in South Korea. Our data provided important insights into new recombination events, which may prove valuable for predicting the emergence of circulating norovirus strains with global epidemic potential.

## Introduction

Viral gastroenteritis is the most common causal agent of public health problems worldwide. Using novel sensitive diagnostic methods, researchers have shown that noroviruses (NoVs) cause acute nonbacterial gastroenteritis in humans of all ages. NoVs are transmitted from person to person through contaminated foods such as raw shellfish and other routes such as aerosols and water [1]. Approximately 23 million cases of gastroenteritis caused by NoVs are estimated to occur in the United States each year, and about 60–85% of all gastroenteritis outbreaks within the United States, Europe, and Japan are associated with NoVs [2], [3].

NoVs comprise a distinct genus within the family *Caliciviridae* and are nonenveloped viruses harboring a positive-sense RNA genome of about 7.7 kb [4]. This genome includes three open reading frames (ORFs): ORF1 encodes nonstructural proteins, including proteases, RNA nucleoside triphosphatases (NTPases), and RNA-dependent RNA polymerase (RdRp), while ORF2 and ORF3 encode the major capsid proteins VP1 and VP2, respectively [5]. Genome characterization studies based on the *RdRp* or *VP1* gene sequences have classified NoVs into five distinct genogroups (GI–GV), which can be further subdivided into 44 genotypes, including the 16 GI, 23 GII, 2 GIII, 2 GIV, and 1 GV genotypes [6]. Recently, a sixth genogroup has been also proposed [7]. The GI, GII, and GIV genotypes are known to infect humans; GII.4 is the most common genotype and has caused global outbreaks and acute gastroenteritis in humans since the mid-1990s [8], [9]. Other genotypes are found less frequently, but have been shown to cause sporadic outbreaks or epidemics [10].

The evolution of new RNA viruses and viral genomes with genetic variation is attributed to genetic recombination, the key mechanism mediating sequence exchange [11]. Viral recombination can change phylogenetic inference, virulence, and epidemiology and can have major implications in vaccination strategies [12]. Several recombinant NoV strains have caused outbreaks of acute gastroenteritis [13], [14], [15]. Additionally, phylogenetic analysis of RdRp- and capsid-encoding genes has supported that NoVs undergo recombination [16]. Predictive models have indicated that common NoV recombinants have breakpoints located either within or close to the ORF1/ORF2 junction. Consequently, this region is thought to constitute a recombination hotspot in NoVs [12].

In South Korea, after rotaviruses, NoVs are the next most important cause of nonbacterial acute gastroenteritis in children. Surveillance studies have indicated that the emergence of NoVs may be associated with both the season and the geographic area. The GII.4 genotype was the most prevalent strain between 2007 and 2008, while reports from 2001 to 2005 showed that GII.1 was the most predominant strain [17], [18].

In this study, we investigated the occurrence of NoV infections in children with acute gastroenteritis at three university hospitals located in South Korea between January 2011 and March 2012. We also analyzed the genetic diversity of NoVs according to genogrouping and genotypes and identified novel recombination events among the identified strains.

## Materials and Methods

### Ethics statement

The stool samples used in this study were collected and analyzed under our protocol (number #2010-10-02), which was approved by the Human Subjects Institutional Review Board (IRB) of Chung-Ang University College of Medicine, Seoul, Korea. For children enrolled in this study, written informed consent was obtained from parents or legal guardians. This consent also included permission to use the data for future research purposes.

### Stool specimens

Stool samples were collected from 502 children hospitalized with acute gastroenteritis at three university hospitals in Seoul between January 2011 and March 2012. All samples were diluted 10-fold with phosphate-buffered saline (pH 7.4) and centrifuged at 10,000*×g* for 15 min. The supernatant was used as the fecal suspension.

### RNA extraction

Viral RNA was extracted using TRI reagent (Gibco BRL Life Technologies, Grand Island, NY, USA), according to the manufacturer's instructions. In brief, 0.3 mL supernatant from fecal suspensions was mixed with 0.7 mL TRI reagent and 0.2 mL chloroform/isoamylalcohol (24∶1). After centrifugation at 12,000×*g* for 15 min, an equal volume of isopropanol was added to the aqueous phase containing RNA. The RNA was precipitated by centrifugation at 12,000×*g* for 10 min, washed with 70% ethanol, dissolved in 20 mL RNase-free water, and stored at −80°C until use in reverse transcription-polymerase chain reaction (RT-PCR).

### RT-PCR

RT was performed with 1-µM concentrations of primer GIR1M for GI capsid or primer GIIR1M for GII [19] and NV35/Reverse for RdRp, as described previously [20]. Nested RT-PCR was performed using the primers listed in Table 1. For capsid genes, the first PCR was performed in a 25-µL reaction tube containing 2 µL cDNA, 2.5 U Ex Taq DNA Polymerase (TaKaRa Bio Co., Shiga, Japan), and 20 pM GIF1M/forward and GIF1M/reverse primers for NoV capsid GI or GIIF1M/forward and GIIR1M/reverse primers for capsid GII. PCR amplification was performed with denaturing at 95°C for 5 min, annealing at 50°C for 1 min, extension at 72°C for 1 min for 30 cycles, and a final extension at 72°C for 10 min. Only the samples were weak or not amplified after the first PCR, the second PCR was performed in a 25-µL reaction containing 1 µL of the first PCR product, 2.5 U Ex Taq DNA polymerase, and 20 pM GIF2/forward and GIR1M/reverse primers for NoV GI capsid or GIIF3M/forward and GIIR1M/reverse primers for NoV GII capsid to generate 307- and 288-bp products, respectively. Thirty-five cycles of PCR amplification were completed under the same conditions as for the first PCR. For the *RdRP* gene, NV32 and NV36 were used as the primers for the first amplification reaction, while NV33 and NV35 primers were used for the second amplification reaction. PCR products were separated on 1.0% SeaKem LE agarose gels (FMC Bioproducts, Rockland, ME, USA) and stained with ethidium bromide. Gels were imaged using a GelDoc XR image-analysis system (BioRad, Hercules, CA, USA).

**Table 1:** Primers used for RT-nested PCR.

Genogroup Region/size	Primer name/polarity	Sequence (5′→3′)	Position a ^,b^	References
Genogroup I Capsid (313 bp)	GIF1M/Forward	CTGCCCGAATTYGTAAATGATGAT	5342–5365	[43]
	GIF2/Forward	ATGATGATGGCGTCTAAGGACGC	5358–5380	[43]
	GIR1M/Reverse	CCAACCCARCCATTRTACATYTG	5649–5671	[43]
Genogroup II RdRp (338 bp)	NV32/Forward	ATGAATATGAATGAAGATGG	4236–4245	[20]
	NV33/Forward	TACCACTATGATGCAGATTA	4280–4299	[20]
	NV35/Reverse	GTTGACACAATCTCATCATC	4597–4617	[20]
	NV36/Reverse	ATTGGTCCTTCTGTTTTGTC	4687–4707	[20]
Capsid (310 bp)	GIIF1M/Forward	GGGAGGGCGATCGCAATCT	5049–5067	[43]
	GIIF3M/Forward	TTGTGAATGAAGATGGCGTCGART	5079–5102	[43]
	GIIR1M/Reverse	CCRCCIGCATRICCRTTRTACAT	5367–5389	[43]

a Norwalk virus for GI (GenBank accession number M87661); ^b^Lordsdale for GII (GenBank accession number X86557).

### Nucleotide sequencing and phylogenetic analysis

Amplified PCR products were cleaned using a QIAquick PCR purification kit (Qiagen, Westburg, Germany) and then sequenced using a BigDye Terminator Cycle Sequencing Kit and an automated DNA sequencer (Model 3730, Applied Biosystems, Foster City, CA, USA). The resulting nucleotide sequences were aligned using CLUSTAL-X 2.1 program with parameters set against the corresponding NoV sequences from the NCBI GenBank database [21]. The nucleotide sequences of the capsid and *RdRp* genes from the study strains were compared with cogent genes of NoVs available in the public database. Phylogenetic trees were constructed using neighbor-joining algorithms from the PHYLIP suite [22] and the Kimura two-parameter model using MEGA6 software. Evolutionary distances for neighbor-joining analyses were based on the model developed by Jukes and Cantor [23]. Tree topology was evaluated using the bootstrap resampling method with 1,000 replicates of the neighbor-joining dataset with the CONSENSE and SEQBOOT programs from the PHYLIP suite. The recombination nucleotide sequences obtained in this study were deposited in the GenBank under the accession numbers KC110854 (4CAU-14), KC10855 (Gil-67), KC110856 (Gil-70), and KC110857 (4CAU-38).

### Recombination analysis

To detect recombination events, an additional RT-PCR was performed using NV33 and GIIR1M primers to amplify a genomic fragment of 1,057 bp, which included the junction between the *RdRP* and capsid genes [24]. The most frequent sites for recombination flanked the capsid region and could be identified using similarity plots in SimPlot software and Bootscan analysis [25], [26]. SimPlot analyzes the percent identity of a query sequence to a panel of reference sequences by position in a stepwise manner across the whole alignment [27]. Identification of recombination events without prior identification of nonrecombinant reference sequences was performed using Bootscan analysis [25].

## Results

### Detection of NoV infections

Of the 502 stool samples, 171 (34%) were positive for NoVs. Of these, the GII genogroup was detected in 170 (99.5%) samples, and the remaining one (0.5%) sample was of the GI.3 genotype. In the GII genogroup, the *RdRp* gene was successful amplified from 123 samples; 117 samples were GII.4, 2 samples were GII.2, 2 samples were GII.16, and 2 samples were GII.b (Table 2). The capsid gene was amplified from 170 samples; 96 samples were the GII.4 genotype, 23 samples were GII.6, 22 samples were GII.12, 20 samples were GII.3, 6 samples were GII.2, 2 samples were GII.b, and 1 sample was GII.10 (Table 2).

**Table 2:** Genotypes of NoVs based on RdRp and capsid genes.

Genogroup	Genotype	Capsid	RdRp
Genogroup I	GI.3	1	-
Genogroup II	GII.2	2	2
	GII.2	4	-
	GII.4	96	117
	GII.6	23	-
	GII.12	22	-
	GII.16	-	2
	GII.3	20	-
	GII.b	2	2
	GII.10	1	-
Total		171	123

### Detection of NoV recombination

Potential NoV recombinant genotypes (*RdRp*/capsid genes) were detected in 54 samples, of which 19 were the GII.4/GII.12 genotype, 16 were GII.4/GII.6, 13 were GII.4/GII.3, two were GII.b/GII.3, two were GII.16/GII.2, one was GII.4/GII.b, and one was GII.2/GII.10 (Table 3).

**Table 3:** Recombination of NoV RdRp/capsid genes.

Genotype	RdRp	Capsid	Number of samples
GII.16/GII.2	GII.16	GII.2	2
GII.2/GII.10	GII.2	GII.10	1
GII.4/GII.12	GII.4	GII.12	12
GII.4/GII.3	GII.4	GII.3	8
GII.4/GII.6	GII.4	GII.6	12
GII.4/GII.2	GII.4	GII.2	1
GII.b/GII.3	GII.b	GII.3	3
Total			39

### Phylogenetic analysis

#### GI genogroup

**Figure 1:**
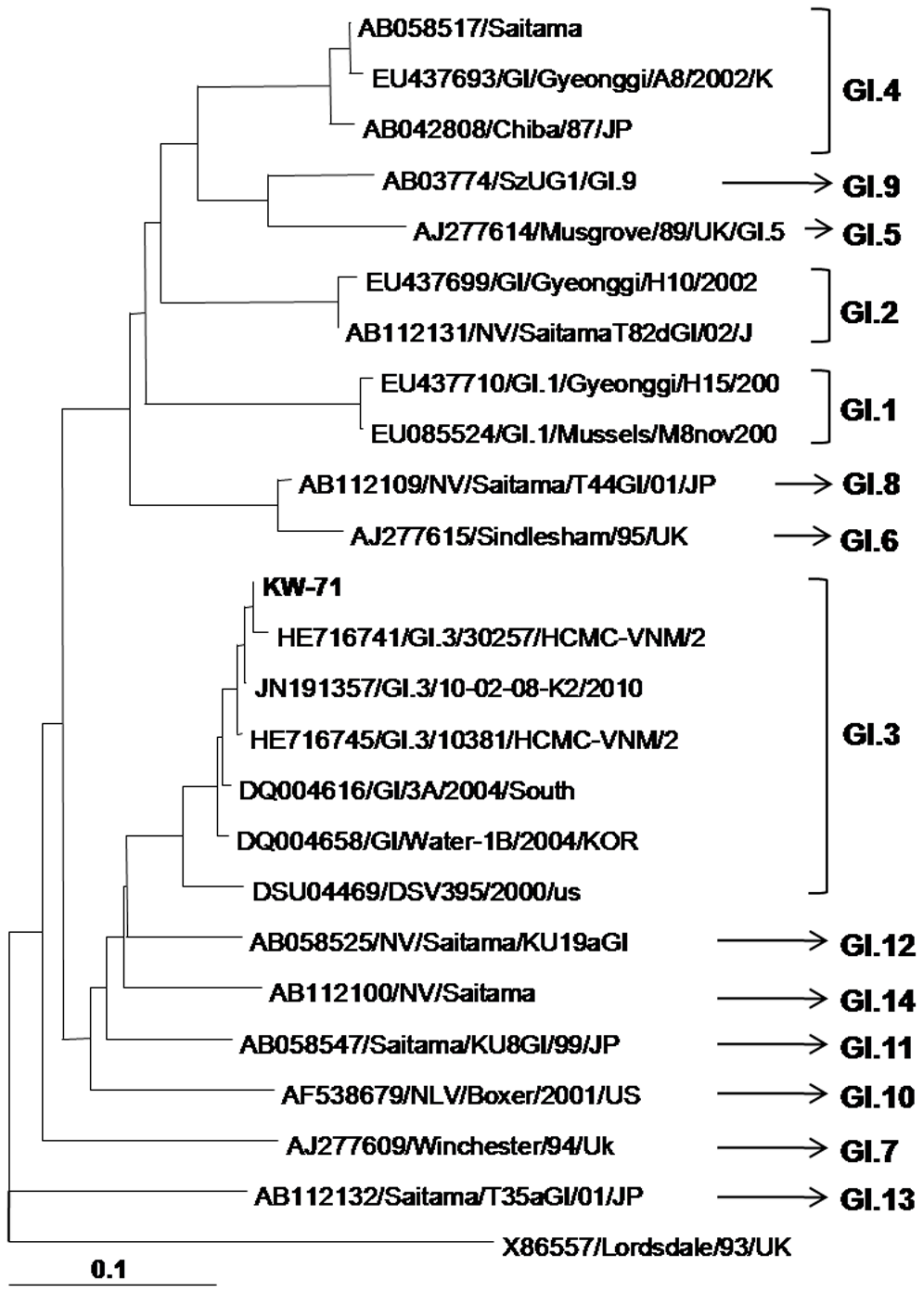
Phylogenetic tree analyse. These analyses involved the partial nucleotide sequences of the norovirus GI strain capsid region detected in this study and the reference strains available in the Genbank database. The scale indicates nucleotide substitutions per position.

The nucleotide sequence of the partial capsid gene (287 bp) of the KW-71 strain was compared with the NoV GI sequences available in the GenBank database. Phylogenetic analysis demonstrated that the capsid gene of the KW-71 strain clustered into the GI.3 genotype (Fig. 1).

#### GII genogroup

**Figure 2:**
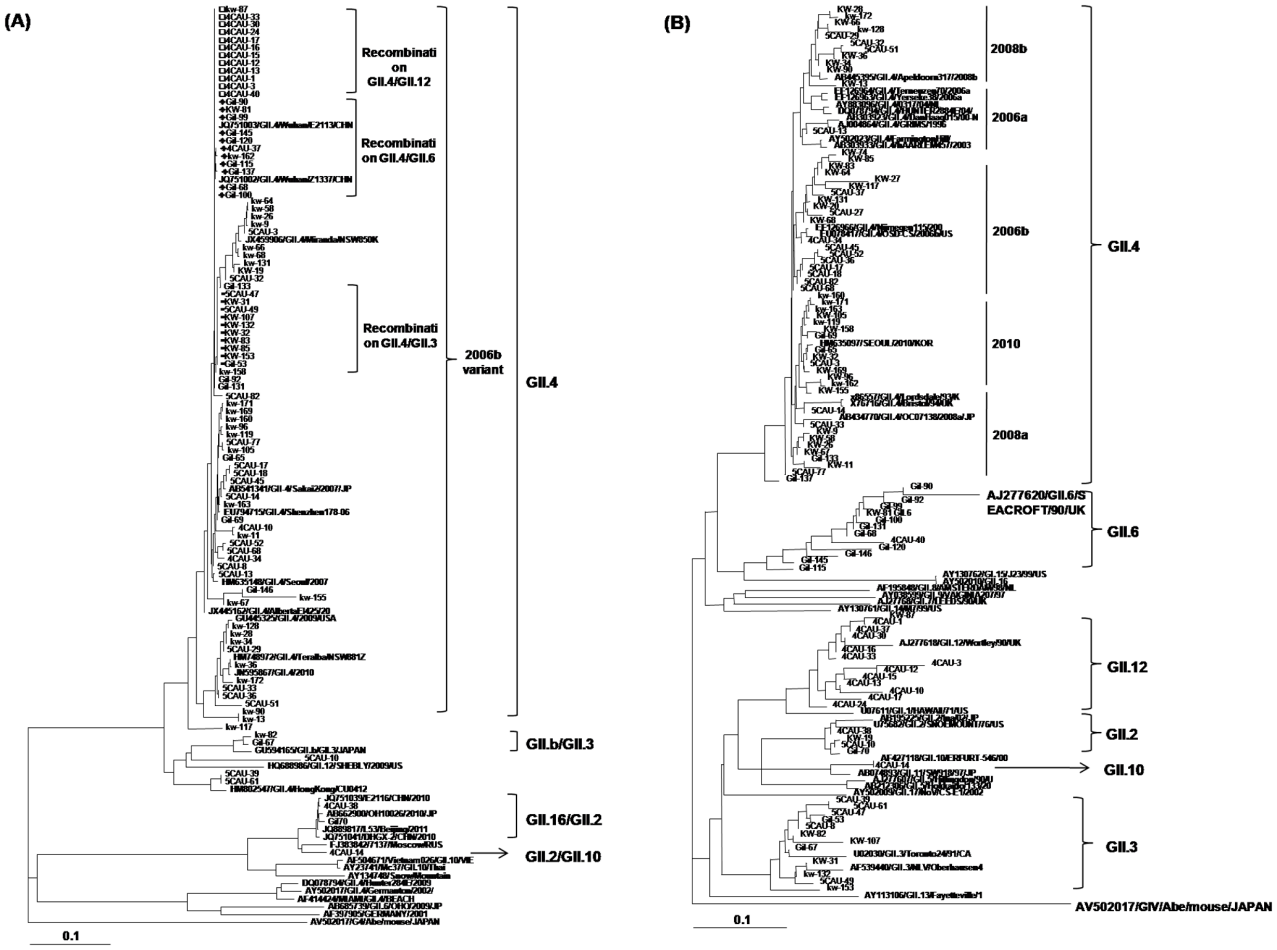
Phylogenetic analyses. These analyses involved the norovirus GII strains detected in this study and the reference strains available in the Genbank database. (A) RdRp gene sequence (328 bp), (B) capsid gene sequence (287 bp). Reference strains are marked in bold type.

he nucleotide sequences of the partial *RdRp* gene (338 bp) and the partial capsid gene (287 bp) from 96 of the 123 NoVs GII strains were determined and compared with those of NoVs available in GenBank. Each sequence data of the remaining 27 samples were subsequently totally identical to one among these 96 strains. The phylogenetic tree showed that most of the *RdRp* and capsid genes from the Korean study strains were classified into the GII.4 genotype and shared nucleotide sequence identities between 94% and 99% (Fig. 2). The most circulated GII strains were the GII.4 2006b variant (56.5%), followed by GII.6 (13.5%), GII.12 (12.9%), GII.3 (11.8%), GII.2 (3.5%), GII.b (1.2%), and GII.10 (0.6%).

In all of the GII.4 strains, the *RdRp* gene belonged to the GII.4-2006b variant. Similar variant strains have also been reported in China (E2113, Z1337, and Shenzhen178-06 strains), Taiwan (NSW850K strain), Japan (Sakai2 strain), South Korea (Seoul strain), Canada (AlbertaEI425 strain), the United States (GU445325 and New Orleans strains), and Australia (NSW881Z strain) and share 97–100% nucleotide sequence identity among themselves and with our GII.4-2006b variant.

Interestingly, the phylogenetic tree revealed that recombination events occurred between the *RdRp*/capsid gene, in which the strain 4CAU-14 belonged to the GII.2/GII.10 recombinant; strains Gil-67, 5CAU10, KW-82 belonged to the GII.b/GII.3 recombinant; strain KW-19 belonged to GII.4/GII.2; strains 5CAU-8, 5CAU-39, 5CAU-47, 5CAU-49, KW-31, KW-107, KW-132, and KW-153 belonged to the GII.4/GII.3 recombinant; strains Gil-90, Gil-92, Gil-99, Gil-100, Gil-68, Gil-131, Gil-120, Gil-146, Gil-145, Gil-115, KW-81, 4CAU-40 belonged to the GII.4/GII.6 recombinant; strains 4CAU-1, 4CAU-3, 4CAU-10, 4CAU-12, 4CAU-13, 4CAU-15, 4CAU-17, 4CAU-16, 4CAU-24, 4CAU-33, 4CAU-30, KW-87 belonged to the GII.4/GII.12 recombinant; and strains 4CAU-38, Gil-70 belonged to the GII.16/GII.2 recombinant (Fig. 2).

### Analysis of recombination events

**Figure 3:**
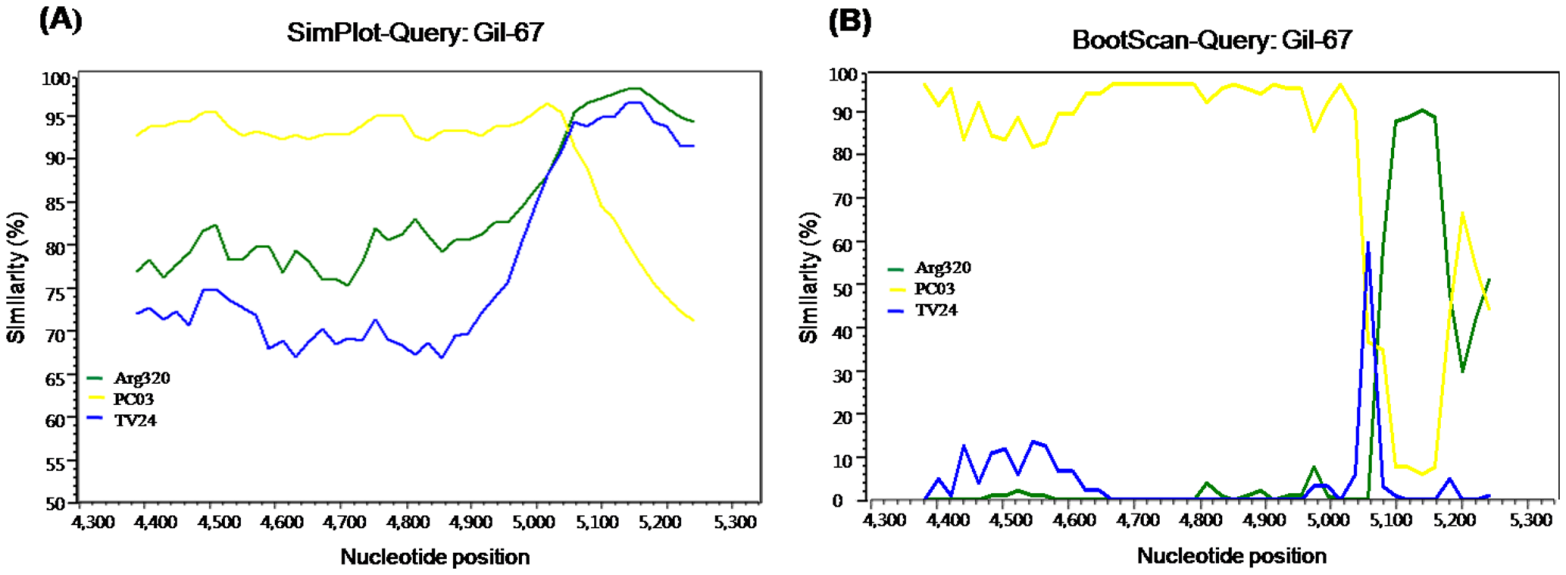
(A) SimPlot analysis of the partial RdRp and capsid gene sequences of the GII.b/GII.3 recombination strain, Gil-67. SimPlot was constructed using Simplot version 3.5 with a slide window width of 200 bp and a step size of 20 bp. The vertical axis indicates nucleotide identities (%) between the query sequence (Gil-67) and the NoVs reference strains. The horizontal axis indicates the nucleotide positions of the analyzed genome regions. **(B) Bootscan analysis of the genomic region involved in the recombination event at position 5,078 of the Gil-67 strain with the NoVs reference strains.**

Recently, reports from several countries have demonstrated that the GII.b/GII.3 recombinant is the predominant pediatric genotype. Phylogenetic analysis indicated that the Gil-67 strain was a GII.b/GII.3 recombinant (Fig. 2). SimPlot software was used to compare the nucleotide sequence of Gil-67 to those of the GII.3 Arg320 strain isolated in Argentina and the GII.b/GII.21 PC03 strain isolated in India. The TV24 strain (U02030) was used as a reference strain where the region of genetic recombination occurred between 5,082 and 5,110 bp at the ORF1/ORF2 overlap region (Fig. 3A) [28]. Upstream of this junction, the nucleotide homology was notably different. Nucleotide sequence comparison showed that the capsid gene of the Gil-67 strain were almost identical with reference strains but shared distinctly different from the *RdRp* gene to the Arg320 strain (data not shown).

Bootscan analysis of Gil-67 was performed to determine the genomic region involved in the recombination event at nucleotide position 5,082 of the PC03 strain as compared to that of the Arg320 strain (Fig. 3B). These strains were suspected to be recombinant NoVs according to the partial capsid and polymerase regions of one representative isolate, the Gil-67 strain. Compared with the Arg320 strain, the Gil-67 strain shared a low level of sequence identity (84%) in the *RdRp* gene region, but higher identity (95%) in the capsid region. In contrast, compared with the PC03 strain, Gil-67 shared a high level of sequence identity (95%) in the *RdRp* gene region and lower identity (65%) in the capsid region. A recombinant site was also observed at the overlap of ORF1 and ORF2 (Fig. 3B).

### Genetic characterization of new recombinant strains

#### GII.16 genotype

**Figure 4:**
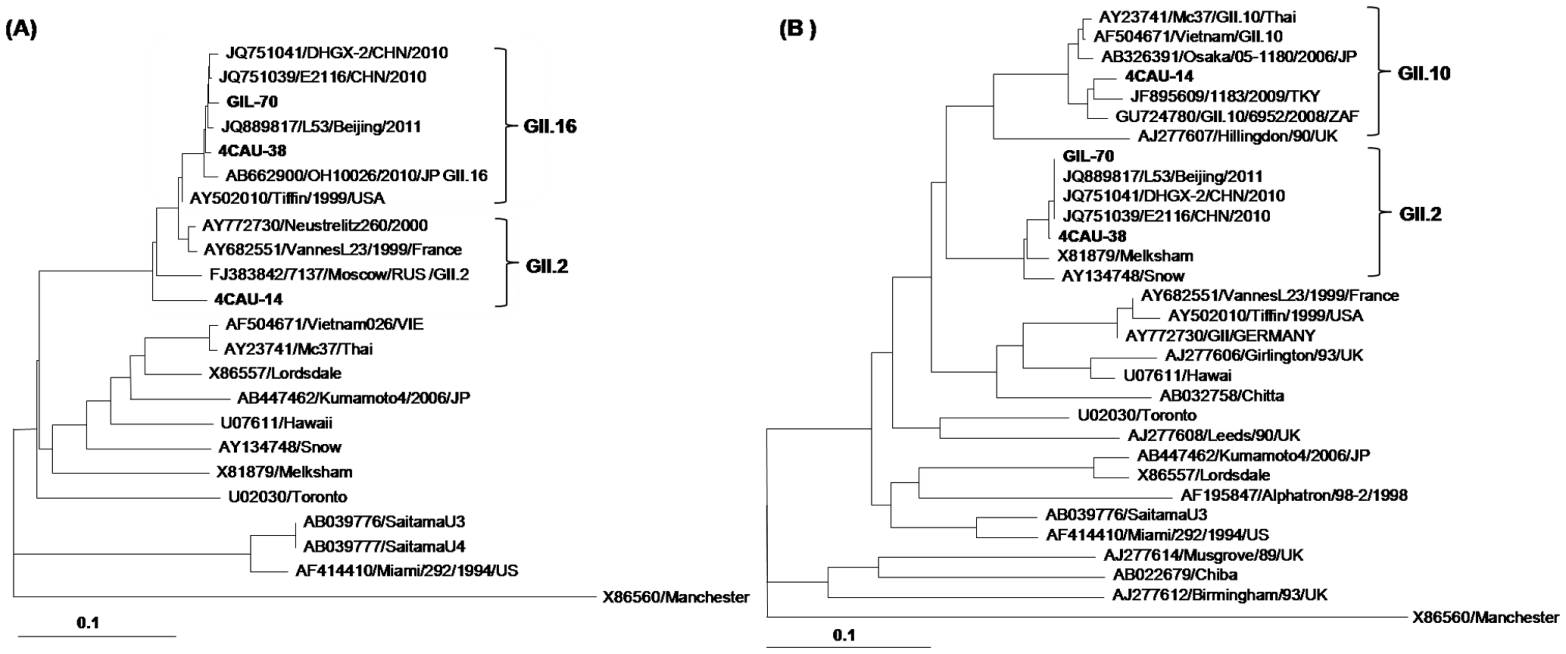
Phylogenetic dendrogram constructed using the neighbor-joining method. The dendrogram was based on (A) the RdRp gene sequences and (B) the capsid gene sequences of the three study strains, Gil-70, 4CAU-38, and 4CAU-14, and of the reference strains available in the Genbank database. Recombinant strains are highlighted in italics.

**Figure 5:**
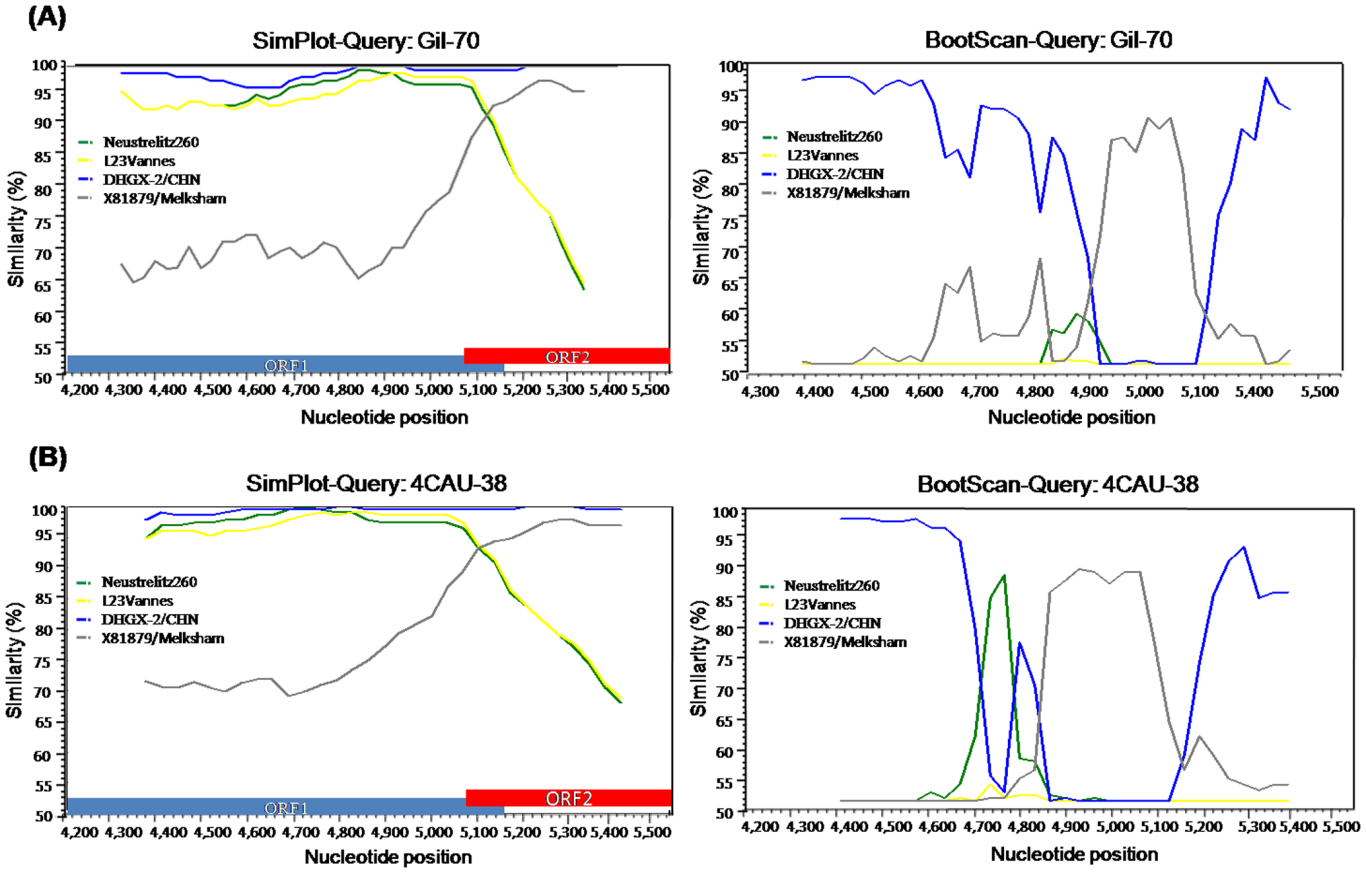
Simplot and Bootscan analysis of the NoVs recombinant GII.16/GII.2, (A) Gil-70 and (B) 4CAU-38 strains. SimPlot was constructed using Simplot version 3.5 with a slide window width of 200 bp and a step size of 20 bp. The vertical axis indicates nucleotide identities (%) between the query sequences (Gil-70 and 4CAU-38) and the reference strains. The horizontal axis indicates the nucleotide positions of the analyzed genomic regions. The vertical line indicates the beginning of ORF2 (5,028 bp) and 20 bp for the ORF1/ORF2 overlap region. Bootscan analysis of the genomic region involved in the recombination event at position 5,028 of the GIL-70 and 4CAU-38 strains with the NoVs reference strains.

Analysis of the full-length nucleotide sequences of the *RdRp* and capsid genes revealed that the Gil-70 and 4CAU-38 strains were GII.16/GII.2 recombinant strains (Fig. 4). The nucleotide sequences of the *RdRp* gene in Gil-70 and 4CAU-38 shared 96.7–99% identity with those of the NoV GII.16 reference strains OH10026, DHGX-2, E2116, and L53. In contrast, the capsid nucleotide sequences of Gil-70 and 4CAU-38 shared 97.2% identity with those of the NoV GII.2 reference strain Melksham (data not shown). SimPlot analysis indicated that the region of genetic recombination of these strains was found between 5,078 and 5,098 bp at the overlap of the ORF1 and ORF2 regions with the GII and GII.16/GII.2 reference strains Melksham, L23Vannes, Neustrelit260, and DHGX-2 (Fig. 5).

#### GII.2 genotype

**Figure 6:**
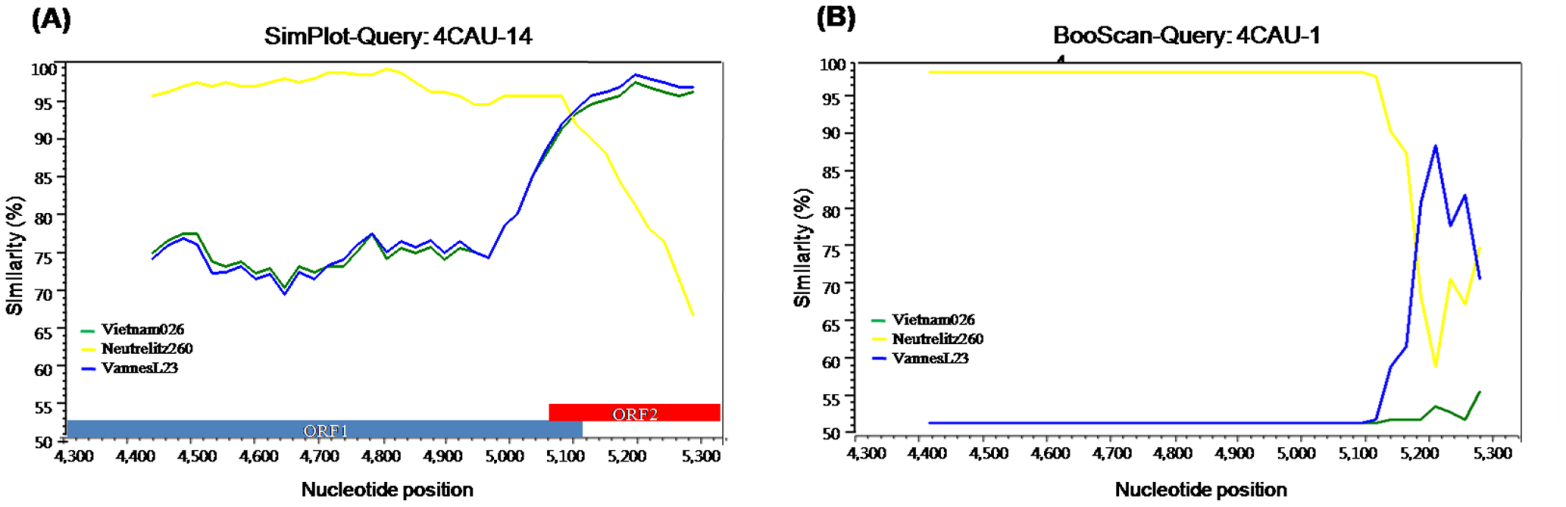
(A) SimPlot analysis of the partial RdRp and capsid gene sequences of recombinant GII.2/GII.10, 4CAU-14 strain. SimPlot was constructed using Simplot version 3.5 with a slide window width of 200 bp and a step size of 20 bp. The vertical axis indicates nucleotide identities (%) between the query sequence (4CAU-14) and the NoVs reference strains. The horizontal axis indicates the nucleotide positions of the analyzed genome regions. **(B) Bootscan analysis of the genomic region involved in the recombination event at position 5,078 of the 4CAU-14 strain with the NoVs reference strains.**

Analysis of the full-length nucleotide sequences of the *RdRp* and capsid genes revealed that the 4CAU-14 strain was a GII.2/GII.10 recombinant strain (Fig. 4). The nucleotide sequence of the *RdRp* gene in 4CAU-14 shared 93.5–100% identity with those of the NoV GII.2 reference strains 7137, Neustrelitz260, and OH08020. In contrast, the nucleotide sequence of the capsid gene in 4CAU-14 shared 95.3–100% identity with those of the NoV GII.10 reference strains 6952, Mc37, and Vietnam026 (data not shown). SimPlot analysis indicated that the region of genetic recombination of these strains was found between 5,078 and 5,098 bp at the overlap of the ORF1 and ORF2 regions with the GII and GII.2/GII.10 reference strains Mc37, Vietnam026, Neustrelitz260, and OH08020 (Fig. 6).

## Discussion

In South Korea, NoV-related gastroenteritis has been a major public health concern since the identification of the virus in 2005, as evidenced by epidemiological studies of the outbreaks that occurred from contaminated groundwater on Jeju Island [18], [29]. Here, we analyzed the prevalence of NoV infections among children hospitalized with acute gastroenteritis at three different localities in South Korea between January 2011 and March 2012. The prevalence of NoV infection in this study was approximately 34%, which was concordant with those of previous reports on NoV epidemiology worldwide [30]–[33]. NoV prevalence ranges from 10% to 60% or even more in pediatric patients hospitalized for acute gastroenteritis, indicating that NoV is an important enteropathogen causing diarrhea in Korean children.

Frequent global outbreaks of NoV-associated acute gastroenteritis have been reported since the late 1990s, and NoV has proven to be the etiological agent in many sporadic cases and outbreaks of gastroenteritis across the globe [34]. To date, three NoV genogroups, GI, GII, and GIV, have been recognized as the main contributors to common outbreaks and are associated with acute gastroenteritis infecting human populations of all ages [35]. In South Korea, while the NoV GII.1 genotype was frequently detected between 2001 and 2005 [17], GII.4 is most predominant genotype reported to date [18], [29]. In this study, the most predominant genotypes were GII.4 (56.5%), followed by GII.6 (13.5%), GII.12 (13%), and GII.3 (11.8%). These data indicated that changes in the patterns of predominant NoV genotypes have occurred over time, suggesting that continuous surveillance is important for early detection and monitoring of the new genotypes in each country.

The occurrence of a variety of different NoVs circulating within a population is a risk factor for recombination events, which in turn can lead to mixed infections [36]. These events strongly impact molecular epidemiological studies, viral vaccine design, and viral control programs [13]. The co-circulation of two potential parental strains may facilitate recombination when the nucleic acid sequences of the strains physically interact in infected cells during copy-choice recombination [37]. RNA recombination is responsible for a large proportion of viral diversity, which is influenced by various immunological and intracellular constraints, leading to the production of viable recombinants [38]. In 1999, Jiang and colleagues were the first to report a naturally occurring NoV recombinant, in which the recombinant site was found between the *RdRp* and capsid genes in the RNA region [16]. Since then, several recombinant strains have been sporadically reported around the globe. Between 2004 and 2007, 21 NoV recombinant types were identified; the NoV recombinant type GII.12/GII.13 was the first isolate identified in South Korea during this period [39]–[41]. In our current phylogenetic analysis of the RdRp/capsid regions, we identified 39 recombinant strains, which were then divided into seven different subtypes: GII.2/GII.10 (n = 1), GII.b/GII.3 (n = 3), GII.4/GII.2 (n = 1), GII.4/GII.3 (n = 8), GII.4/GII.6 (n = 12), GII.4/GII.12 (n = 12), and GII.16/GII.2 (n = 2). The NoV GII.16/GII.2 recombinant, reported in South Korea for the first time in this study, was the predominant strain in Japan during the 2009–2010 season [42]. Interestingly, the NoV GII.2/GII.10 recombinant (4CAU-14 strain) could be characterized as a novel intergenotype recombinant, which highlights the genetic variation in the *RdRp*/capsid genes among strains that have been circulating across the country. Therefore, understanding the recombination events occurring in NoV strain is important for tracking the emergence of new NoV variants [40].

Similarity Plots, otherwise known as SimPlots, can be used to recognize the percent similarity between the query sequences and a panel of reference sequences. Recombinant breakpoints were found between the overlap of ORF1 and ORF2 at nucleotide positions 5,078 and 5,098 bp for the GII.16/GII.2 (Gil-70 and 4CAU38) new recombinant strains and at nucleotide position 5,082 and 5,110 bp for the GII.b/GII.3 (Gil-67) recombinant strain. As seen in other recombinant NoVs, the results from the current study indicate that the region between the ORF1 and ORF2 junction may play a role in recombination events [27].

The findings of our present study support the hypothesis that certain NoV strains circulate in specific areas without clinical manifestations and may occasionally change their genetic properties through recombination events. Since recombination allows a virus to increase its genetic fitness, evolve, spread among the population, and probably escape the host immune response, our findings suggest that NoVs have a huge capacity for genetic change, which will facilitate the generation of new recombinants. NoV infections continue to be important gastroenteritis-associated pathogens infecting the Korean population, with GII.4-2006b variants currently being the most predominant strains. Here, we report the first finding of NoV GII.16/GII.2 recombinants in Korea, with GII.2/GII.10 being the first recombinant described thus far. Continuous surveillance is required to understand the continuously evolving status of NoVs infections in the Korean population.
